# A suitable sampling strategy for the detection of African swine fever virus in living and deceased pigs in the field: a retrospective study

**DOI:** 10.3389/fvets.2024.1419083

**Published:** 2024-06-26

**Authors:** Xiaowen Li, Zhiqiang Hu, Xiaogang Tian, Mingyu Fan, Qingyuan Liu, Xinglong Wang

**Affiliations:** ^1^College of Veterinary Medicine, Northwest A&F University, Xianyang, China; ^2^Shandong Engineering Laboratory of Pig and Poultry Healthy Breeding and Disease Diagnosis Technology, Xiajin New Hope Liuhe Agriculture and Animal Husbandry Co., Ltd., Dezhou, China; ^3^China Agriculture Research System-Yangling Comprehensive Test Station, Xianyang, China; ^4^College of Animal Science, Xichang University, Xichang, China

**Keywords:** African swine fever, mandibular lymph nodes, tonsil, qPCR, deceased and live pigs

## Abstract

African swine fever (ASF) is a fatal disease that threatens the health status of the swine population and thus can impact the economic outcome of the global pig industry. Monitoring the ASF virus (ASFV) is of utmost concern to prevent and control its distribution. This study aims to identify a suitable sampling strategy for ASFV detection in living and deceased pigs under field conditions. A range of samples, comprising tissues obtained from deceased pigs, as well as serum and tonsil swab samples from live pigs, were gathered and subjected to detection using the qPCR method. The findings revealed that the mandibular lymph nodes demonstrated the highest viral loads among superficial tissues, thereby indicating their potential suitability for detecting ASFV in deceased pigs. Additionally, the correlations between virus loads in various tissues have demonstrated that tonsil swab samples are a viable specimen for monitoring live pigs, given the strong associations observed with other tissues. These findings indicated two dependable sample types for the detection of ASFV: mandibular lymph nodes for deceased pigs and tonsil swabs for live pigs, which supply some references for the development of efficacious preventive measures against ASFV.

## Background

African swine fever (ASF) is a contagious disease caused by the African swine fever virus (ASFV), capable of infecting diverse porcine species. ASFV, belonging to the *Asfivirus* genus within the *Asfarviridae* family, is an enveloped double-stranded DNA virus with a diameter of approximately 200 nm, and is also the only DNA arthropod-borne virus discovered to date (1, 2). The first description of ASFV was done in Kenya in 1921, and then introduced from Africa to Portugal occurred in 1957, subsequently leading to outbreaks in various European countries (3–5). Notably, in 2018, China experienced its first outbreak in Shenyang City, which rapidly spread throughout the nation, posing a grave threat to the domestic pig industry (6). The transmission and excretion patterns of ASFV strains with different virulence were different in domestic pigs. The advent of attenuated strains of ASFV has resulted in a gradual reduction of clinical symptoms, leading to significantly reduced viral loads in blood, saliva, and feces, and in some cases, the virus is completely undetectable by existing testing methods in these above sample types (7–9). Consequently, monitoring clinical manifestations becomes increasingly challenging.

Research has demonstrated that highly virulent ASFV strains typically require an incubation period in pigs for 9–12 days before they can be detected in the blood, whereas less virulent strains exhibit lower viral loads and intermittent clearance upon detection (10, 11). Moreover, the duration required for virus detection in the bloodstream varies depending on the various modes of infection, with contact transmission exhibiting a delay of 1–2 days compared to intramuscular injection (8, 11, 12). Additionally, ASFV has been reported to be able to spread through multiple routes, including contact and aerosol transmission (11, 13, 14). And also, ASFV-positive pigs have been found to harbor a significant quantity of virus particles in their blood and deep tissues (10, 11, 15–17), which indicates that the process of blood sampling and necropsy procedures may lead to extensive contamination of the virus within the facility in the event of ASFV outbreaks in large-scale farms. Consequently, the accurate evaluation of ASFV presence in appropriate clinical samples is crucial for mitigating ASFV transmission in extensive commercial pig operations (18). This study aims to compare viral loads across various tissues, thereby establishing a benchmark for ASFV detection and early warning systems.

## Materials and methods

### Sample source

All the samples consisted of clinical disease materials that had been accumulated and preserved at a temperature of −80°C in our laboratory over the past 3 years. The tissue samples were obtained from 23 suspected dead-ASFV positive pigs, which were collected by trained veterinarians and subsequently sent to our laboratory for qPCR testing within 24 h of sample collection. Each tissue sample of 0.02 g was subjected to grinding using a tissue grinder, and subsequently mixed with 1 mL of PBS. Serum samples (*N* = 74) and tonsil swab samples (*N* = 74) were acquired from live pigs by trained veterinarians in several ASFV-positive herds undergoing ASF precision culling, and sent to our laboratory for testing within 24 h of sample collection. In case of serum collection, 5 mL of blood was extracted from the anterior vena cava of each pig, from which 1–2 mL of serum was isolated. Tonsil swab sampling involved the insertion of swabs into the deep tonsil position of pigs, which were then held for a minimum of 3 s, removed, and subsequently dissolved in 3 mL of normal saline.

### qPCR

All the samples were tested using qPCR following the previously described method (19). Briefly, 300 μL of serum or throat swab eluent were subjected to DNA extraction using the Automatic nucleic acid extractors (NPA-96E) from Bioer Technology Co., Ltd. (Hangzhou, China). Subsequently, 5 μL of the extracted DNA was utilized for qPCR detection, which was performed on a Step One Plus instrument (ABI) using the PerfectStart^®^ II Probe qPCR SuperMix (TransGen Biotech, China) according to the manufacturer’s instructions. Specific primers for the ASFV B646L gene were designed based on the ASFV isolate Pig/HLJ/18 (GenBank: MK333180.1) (18) and used for qPCR: 5’-AAAATGATACGCAGCGAAC-3’(forward), 5’-TTGTTTACCAGCTGTTTGGAT-3’ (reverse), and 5’-FAM-TTCACAGCATTTTCCCGAGAACT-BHQ1-3’ (probe) (17). The results of qPCR were recorded as quantification cycle values (Cq values), and a Cq value of <40 was considered as a positive result. The limit of detection (LOD) of this method is 2.5 copies/μL (19), which is more sensitive than the method recommended by World Organization for Animal Health (WOAH) (20).

### Statistical analyses

The mean copy numbers among different tissues were compared using the paired t-test in the GraphPad Prism software (version 8.0). The Pearson correlation analysis between different tissues was performed to construct the correlation coefficient matrix and the relation coefficient between serum and tonsil swab samples using the GraphPad Prism software (version 8.0) as well. A *p* value of <0.05 was considered to be statistically significant.

## Results

### Viral loads in different tissues

**Figure 1:**
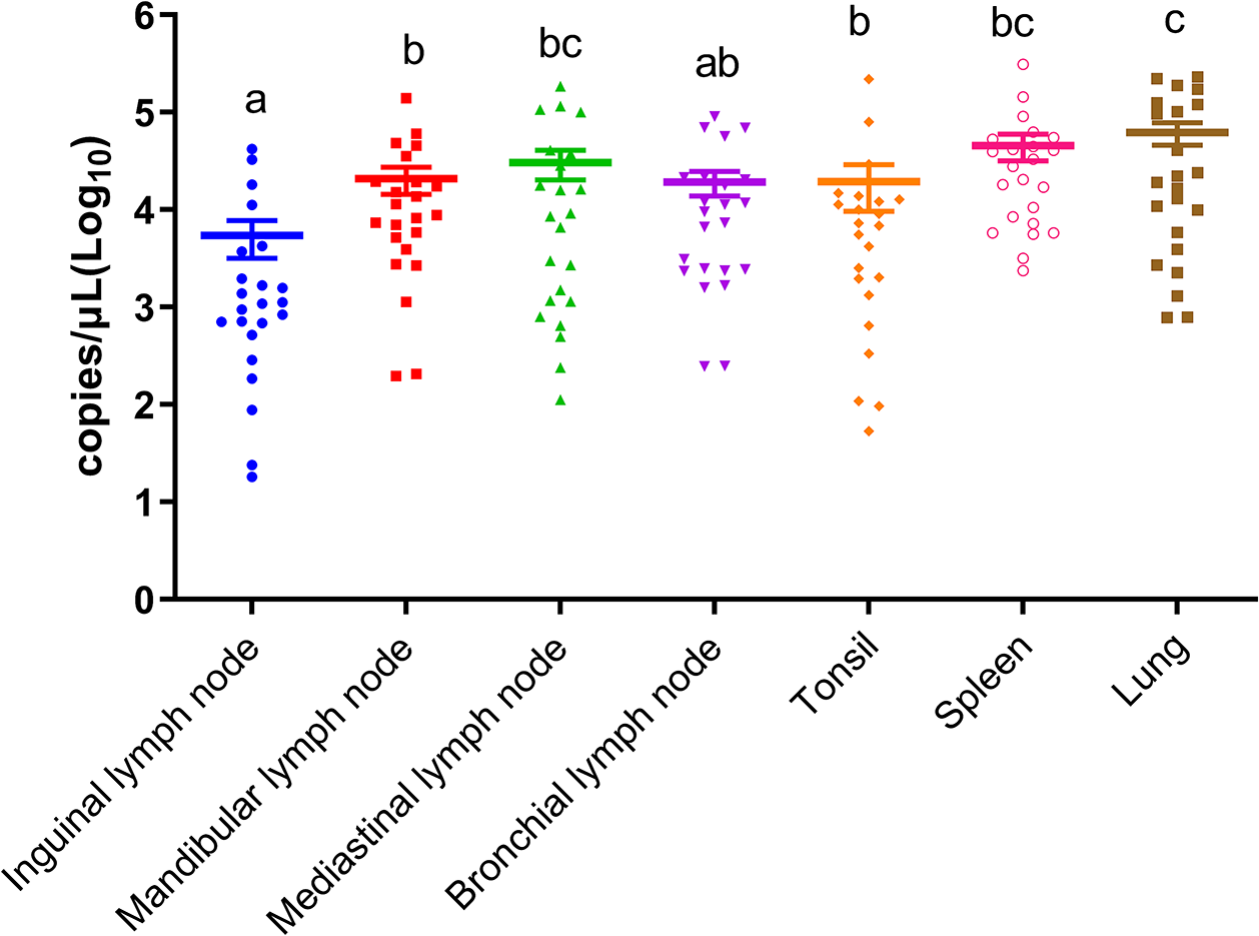
Viral loads of ASFV in inguinal lymph nodes, mandibular lymph nodes, mediastinal lymph nodes, bronchial lymph nodes, tonsils, spleens, and lungs. Different letters indicate significant statistical differences (*p* < 0.05), and the same letter indicates no significant statistical differences (*p* > 0.05).

To identify the distribution of the virus in different tissues of deceased pigs, a comparison of viral loads in various tissues from the same pig were conducted. As shown in Figure 1, there was a descending order of viral loads from high to low: lung, spleen, mediastinal lymph nodes, mandibular lymph nodes, bronchial lymph nodes, tonsils, and inguinal lymph nodes. Notably, in superficial tissues, the viral loads in mandibular lymph nodes and tonsils were significantly higher than those in inguinal lymph nodes (*p* < 0.05), suggesting that mandibular lymph nodes are a viable option for detecting ASFV under field conditions.

### Correlation analysis between different tissues

**Figure 2:**
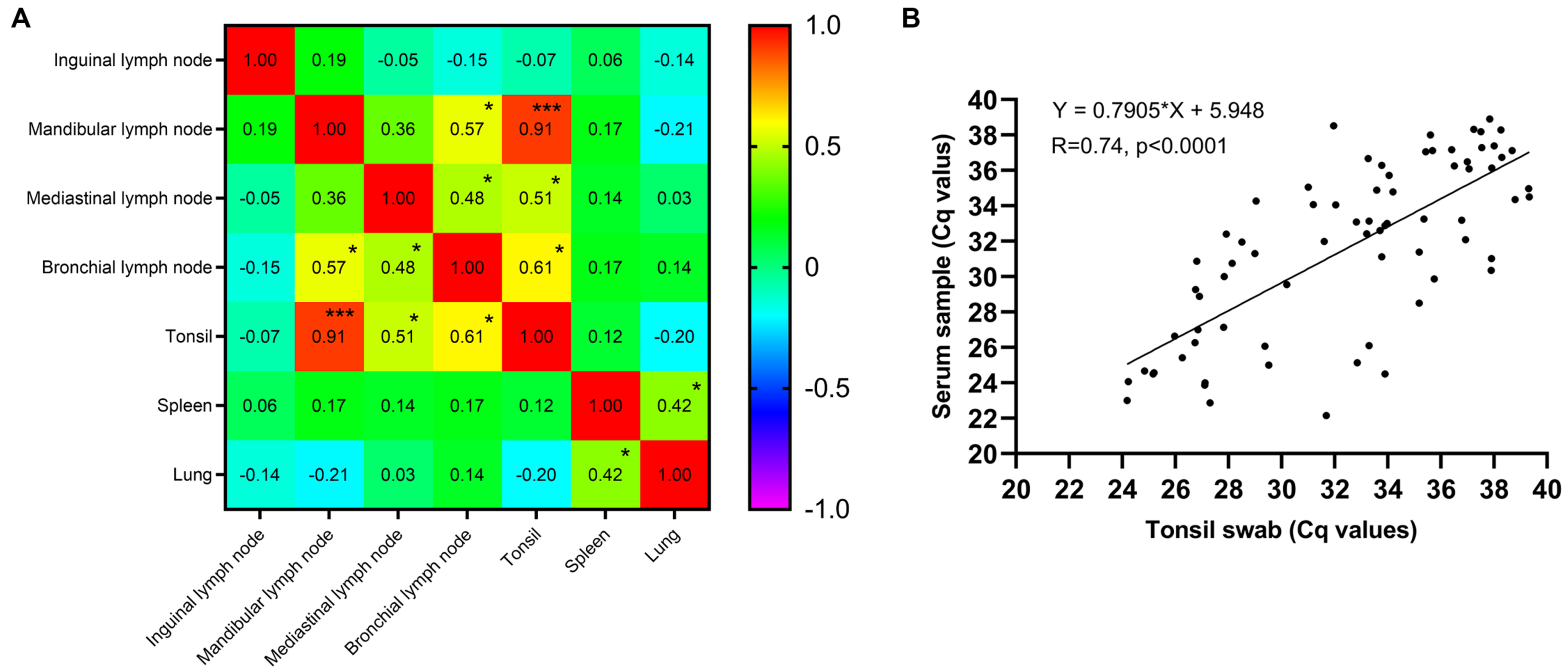
**(A)** The correlation coefficient matrix of ASFV genome copies between different tissues. *p value <0.001, ***p value <0.001. **(B)** The correlation coefficient between serum and tonsil swab samples.

Through the examination of virus load correlations across various tissues, as shown in Figure 2A, the investigation revealed that the most substantial correlation (*R* = 0.91, *p* < 0.001) was observed between tonsils and mandibular lymph nodes, followed by the correlations between tonsils and bronchial lymph nodes (*R* = 0.61, *p* < 0.05). Furthermore, a significant correlation was also identified between tonsils and mediastinal lymph nodes (*R* = 0.51, *p* < 0.05). These findings suggest that tonsils may serve as a suitable choice for sample collection in pigs. Moreover, a total of 74 positive pigs were subjected to the collection of serum and tonsil swab samples, and their correlation was subsequently analyzed. The findings shown in Figure 2B demonstrate a correlation coefficient (*R* value) of 0.74 between tonsil swabs and serum samples, suggesting that tonsil swabs possess the potential to serve as a viable alternative to serum samples for the detection of ASFV in live pigs.

## Discussion

ASF is a profoundly deleterious ailment, exhibiting an almost absolute fatality rate. Its clinical progression encompasses hyper-acute, acute, sub-acute, and chronic phases (21). The incubation period of ASFV varies, contingent upon the distinct strains, modes of infection, and the immunological status of the host pig, typically spanning from 3 to 19 days (22–24). Pigs infected with highly virulent or moderately virulent strains exhibit an elevated mortality rate, and the virus disseminates within the porcine population even prior to the manifestation of clinical symptoms (11). Hence, it is crucial to promptly detect and ascertain the presence of ASFV infection in pig populations to effectively manage and mitigate the losses incurred by ASFV outbreaks (25).

Presently, ASFV surveillance is predominantly carried out using two approaches: passive and active monitoring. Passive monitoring primarily entails the surveillance of deceased pigs. Sample types for ASFV detection in deceased pigs, as recommended by WOAH, include spleen, lymph nodes, bone marrow, lung, tonsil and kidney (16, 26). Clinical sample types that have been successful in isolating ASFV strains primarily consist of the spleen, lymph nodes, lung, and blood (10, 15). Additionally, literatures have reported comparisons of viral loads in various organs of ASFV-infected animal models (10, 15), revealing that viral loads in the spleen, lungs, tonsils, and lymph nodes of ASFV-positive pigs were consistently high and stable. A systematic investigation was undertaken to examine 11 organs from 10 pigs, which unveiled that livers exhibited the highest viral loads, followed by spleens and inguinal lymph nodes, indicating ASFV causing immunosuppression in the host (27, 28). ASFV has been documented to possess various mechanisms to evade or dismantle the host’s immune system (29). Hence, immune-related organs persist as the primary sites for the accumulation of ASFV particles. Several investigations have indicated the presence of ASFV in mandibular lymph nodes, mediastinal lymph nodes and bronchial lymph nodes of pigs infected with low-virulence ASF strains (10, 15, 30). Given the aforementioned rationales, in order to compare the differences in viral loads between superficial tissues and deep tissues of naturally ASFV-infected deceased pigs, representative deep tissues such as spleens, lungs, mediastinal lymph nodes, and bronchial lymph nodes, along with representative superficial tissues like tonsils, inguinal lymph nodes, and mandibular lymph nodes, were selected for detection and analysis. The findings of the study revealed that virus loads were significantly higher in deep tissues, specifically in the lungs and spleens. Conversely, in superficial tissues (tonsils, inguinal lymph nodes, and mandibular lymph nodes), the mandibular lymph nodes exhibited the highest virus loads, ranking second only to the lungs and spleens. However, obtaining these deep tissues requires opening up the carcasses, which often leads to contamination of the premises (16). Therefore, it is advisable to prioritize the collection of samples from superficial organs to ensure safe and reliable results. Pikalo et al. studied the potential of multiple superficial sample types in the passive detection of ASFV, including ocular fluids, superficial lymph nodes and ear punches, and demonstrated that superficial lymph nodes and ear punches could be potential alternatives (28). Additionally, inguinal lymph nodes have traditionally been regarded as a suitable tissue for monitoring deceased pigs, as they allow for minimally invasive sampling and minimize the risk of contamination (16, 19). This study revealed a noteworthy disparity in viral loads between mandibular and inguinal lymph nodes, with mandibular lymph nodes exhibiting significantly higher levels, which was consistent with the finds of Pikalo’s (28). The mandibular lymph node is located in the mandibular space, on the inside of the lower margin of the left or right mandibular angle, in front of the submandibular gland, and is covered by the oral end of the subauricular gland (31). Furthermore, a lymph node sampler developed by our laboratory can also be used to sample mandibular lymph nodes in dead pigs (19). Consequently, this finding suggests that mandibular lymph nodes may prove to be a more suitable option for the screening of deceased pigs for ASFV in subsequent investigations.

Active monitoring primarily entails the surveillance of live pigs, typically encompassing the acquisition of serum samples, as there are a large number of virus particles in the blood in ASFV-positive pigs (11, 13, 14). Moreover, given the intricate nature of blood sampling process in pigs, the involvement of a minimum of 2–3 individuals is necessary, potentially heightening the risk of viral transmission and broadening the extent of pathogen contamination on the premise (32). Consequently, it becomes imperative to explore alternative sample types that can substitute serum samples while being easily obtainable. Several alternative blood samples have been evaluated, including oral swabs, rectal swabs, nasal swabs, feces and so on (15, 17, 19). Research has demonstrated that the genome of ASFV can be detected in the oral fluids of weaned pigs infected with highly virulent ASFV Georgia 2007/1 and moderately virulent ASFV Malta’78 strains within a period of 3–5 days after infection (33). This study has revealed that tonsils exhibit a relatively strong correlation with other tissues, and a strong correlation (*R* = 0.74) between tonsil swabs and serum samples, thus suggesting that tonsil swabs from tonsil exudate could serve as a suitable alternative sample for diagnosing ASF infection. The utilization of tonsil swabs as opposed to serum samples for collection purposes offers advantages in terms of time and cost efficiency. Our laboratory has developed a tonsil swab sampler that uses a unique short fiber villus material to absorb as much tonsil exudate as possible. When using, the long swab is inserted into the deep position of tonsils, held for a minimum of 3 s, removed, and subsequently dissolved in normal saline, which is easy to operate, and no additional assistance tools or farmers to fixed pigs are required. This tool has been widely used in farms in China. Nevertheless, it is crucial to take into account the presence of diverse ASFV strains with varying levels of virulence when monitoring tonsil swab samples. Further research is imperative to ascertain the reliability and precision of this method in monitoring pigs infected with lower virulent strains.

In summary, based on the comparison of viral loads between different tissues of deceased pigs and between different sample types of live pigs, this study identified mandibular lymph nodes as a preferable superficial tissue sample choice for screening deceased pigs, while tonsil swabs were found to be a viable alternative for diagnosing ASF infections in live pigs. However, the findings of this study are only based on samples from field conditions. More in-depth and accurate study requires more researchers and laboratories to explore in the future. Our findings provide a good laboratory research direction, and also have the potential to provide valuable insights for enhancing the efficacy of clinical prevention and control strategies against ASFV outbreaks, thereby aiding in the mitigation of associated economic losses.

## Data availability statement

The raw data supporting the conclusions of this article will be made available by the authors, without undue reservation.

## Ethics statement

Ethical approval was not required for the study involving animals in accordance with the local legislation and institutional requirements because all the samples were clinical samples provided by pig farms for ASFV diagnostic detection. Then, these clinical samples were accumulated and stored in our laboratory, therefore this is a retrospective study. Written informed consent was obtained from the owners of the animals to use the clinical samples.

## Author contributions

XL: Formal analysis, Writing – original draft, Writing – review & editing. ZH: Formal analysis, Writing – original draft, Writing – review & editing. XT: Data curation, Formal analysis, Software, Writing – original draft. MF: Data curation, Formal analysis, Software, Writing – original draft. QL: Data curation, Formal analysis, Software, Writing – original draft. XW: Resources, Writing – review & editing.
